# The Great COVID-19 Divergence: Managing a Sustainable and Equitable Recovery in the EU

**DOI:** 10.1007/s10272-021-0983-8

**Published:** 2021-08-05

**Authors:** Grégory Claeys, Zsolt Darvas, Maria Demertzis, Guntram B. Wolff

**Affiliations:** Bruegel, Rue de la Charité 33, 1210 Brussels, Belgium

## Abstract

The COVID-19 pandemic has led to the biggest global recession since the Second World War. Forecasts show the European Union underperforming economically relative to the United States and China during 2019–2023. Southern European countries have been particularly strongly affected. Some sectors have been hit harder than others. Business insolvencies have, paradoxically, fallen. While total employment has almost recovered, the young and those with low-level qualifications have suffered employment losses. Inequality could rise. The pandemic may lead to lasting changes in the economy, with more teleworking, possibly higher productivity growth and changed consumer behaviour. Policymakers must act to prevent lasting divergence within the EU and scarring due to the fallout from the pandemic. The first priority is tackling the global health emergency. Second, the article warns against premature fiscal tightening but suggests additional short-term support to prevent scarring. Third, the article warns against protectionism and advocates for reforms that boost productivity growth further.

The COVID-19 pandemic has led to the biggest global recession since the Second World War. Global GDP in 2020 was 6.7% lower than had been forecast at the end of 2019.[1] Developing and advanced countries lost about the same proportion of output relative to the forecast (6.7% vs 6.3%), yet the actual annual GDP decline was larger in advanced economies: a 4.7% recession in 2020 vs a 2.2% recession in 2020 in emerging and developing countries respectively. Among the big economies, China even grew by 2.3%, though its 2020 level of GDP was 3.6% lower than pre-pandemic forecasts.

According to the International Monetary Fund (IMF, 2019, 2021), despite higher-than-usual growth as the global economy recovers from the COVID-19 shock, world output will still be about 4% lower in 2024 than pre-pandemic projections suggested. In other words, the global economy looks set to suffer from longer-lasting scarring effects that could permanently lower the path of output.

Within the European Union, some countries have seen greater GDP losses than others. Some sectors have been harder hit than others, and there have been different impacts on the labour market depending on age, gender and education level. These differences are documented in the following section.

Some of the intra-EU divergence may become permanent or at least long lasting, as discussed in the subsequent section. For example, GDP forecasts suggest that some countries, such as Italy, will reach their pre-pandemic GDP level only by 2023 while others, such as Poland, will

* This Paper was prepared for the informal EU Economic and Financial Affairs Council (ECOFIN) meeting in Lisbon on 21-22 May 2021. The authors are grateful to Monika Grzegorczyk, Lionel Guetta-Jeanrenaud, Mia Hoffmann, Klaas Lenaerts, Ben McWilliams, Tom Schraepen and Pauline Weil for excellent research assistance and to Mario Mariniello, Simone Tagliapietra, André Sapir and Nicolas Véron for their useful comments. All remaining errors are our own.

1 Based on a comparison of the October 2019 and the April 2021 World Economic Outlook forecasts (IMF, 2019, 2021).

**Grégory Claeys**, Bruegel, Brussels, Belgium.

**Zsolt Darvas**, Bruegel, Brussels, Belgium; and Corvinus University of Budapest, Hungary.

**Guntram B. Wolff**, Bruegel, Brussels, Belgium.

Figure 1

**Real GDP forecasts as of April 2021: Cumulative growth for 2019-2023**

in %

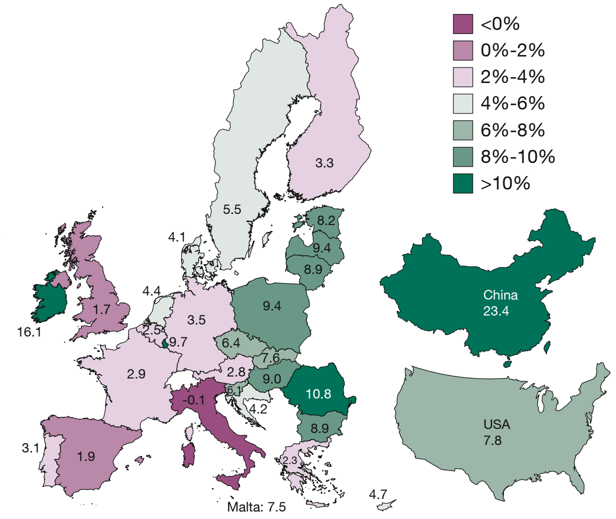

Note: Forecast EU27 cumulative growth for 2019-2023 is 4.1%. Countries in shades of purple are thus below the EU27 average, while those in green are above. Irish GDP numbers reflect the large role of foreign multinationals and should therefore be considered with care.

Source: Authors' own calculations based on IMF (2021).

Figure 2

**Change in real GDP growth forecasts between October 2019 and April 2021 for 2019-2023**

in percentage points

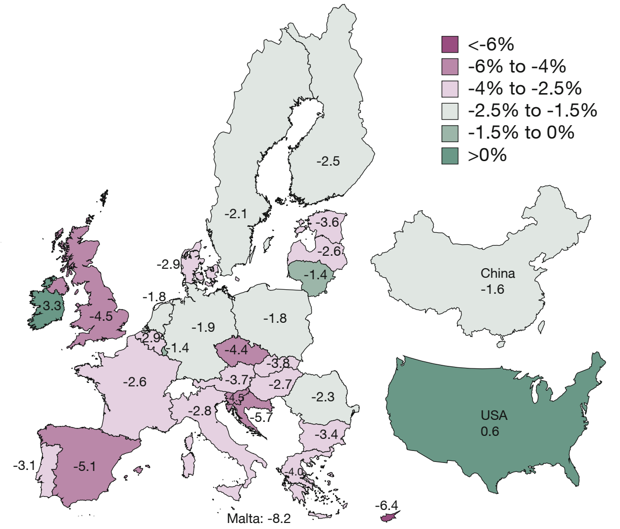

Note: The difference between the October 2019 and the April 2021 forecasts for the 2019-2023 EU27 growth is -2.5 percentage points. Countries in shades of purple are thus below the EU27 average, while those in green are above.

Source: Authors' own calculations based on IMF (2019, 2021).

surpass it in 2021 already.[2] On a sectoral level, the pandemic might lead to a different economy because of long-lasting behavioural changes.

As the EU emerges from the COVID-19 recession, important policy choices need to be made to prevent unnecessary long-term damage, facilitate the necessary sectoral reallocation, address the inequality effects of the pandemic and ensure a sustainable recovery. We analyse these choices in the final section.

## EU divergence

According to current forecasts, from 2019 to 2023, the EU economy is set to underperform relative to that of the United States and China. There will also be divergence within the EU. Figure 1 shows expected cumulative growth over this period, highlighting the economic underperformance of large parts of the EU relative to the US and China, and of countries in the Mediterranean and of the United Kingdom.

The coronavirus pandemic has been one of the main drivers of this underperformance. Figure 2 shows that growth forecasts for the period 2019-2023 have been strongly revised downwards in some countries in the south of Europe, in the Czech Republic and in the UK during the pandemic, while forecasts for 2023 for the US and Ireland have actually improved compared to pre-crisis forecasts.

Multiple factors can explain this picture. Sapir (2020) suggested that the differential impact of the pandemic on economic performance can be explained by the strictness of the lockdowns necessary to contain the pandemic, the size of countries' tourism sectors and the overall quality of their governance.

Clear sectoral divergences can be seen, with the tourism sector, and the services sector more broadly, particularly affected. Figure 3 shows the stark differences between sectors. It also shows that most sectors were able to reorganise so that, compared to the first lockdown, the second lockdown in the fourth quarter of 2020 affected

2 There are different dates for the return to pre-pandemic level of output depending on whether we use annual or quarterly data. In this article, we mostly rely on the April 2021 IMF forecast of annual data, because that is available up to 2026, while the May 2021 European Commission forecast is available only up to 2022. For 2021-2022, European Commission forecasts are slightly more optimistic than those of the IMF, yet the Commission forecasts reflect similar cross-country differences as the IMF forecasts. The Commission also presents quarterly forecasts. For Italy, the Commission's quarterly forecast suggests that output will return to its pre-pandemic level by the end of 2022, yet the Commission's annual forecast indicates that Italian GDP in 2022 will not yet reach the annual 2019 value.

Figure 3

**Gross value added in selected sectors, EU27**

index, 2019Q1=100

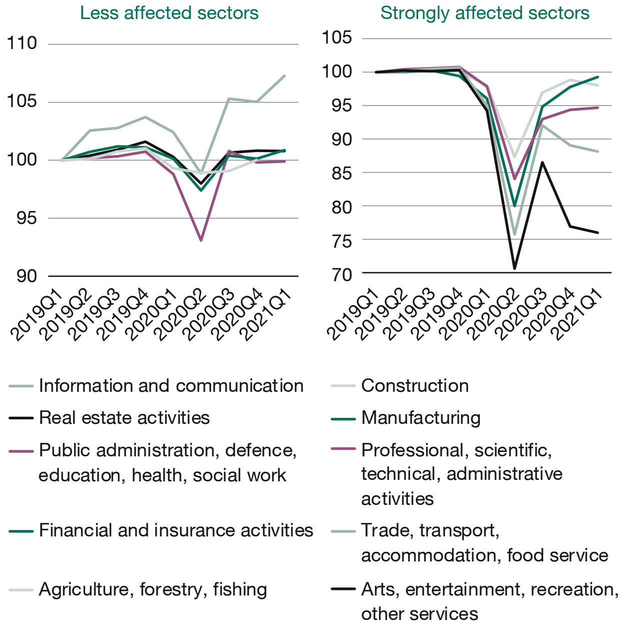

Note: Chain-linked volumes, seasonally adjusted.

Source: Authors' own calculations based on Eurostat's "Gross value added and income A*10 industry breakdowns [namq_10_a10]" dataset, 2021.

Figure 4

**Business insolvency filings during the global financial crisis and the COVID-19 crisis**

index, pre-recession quarter = 100

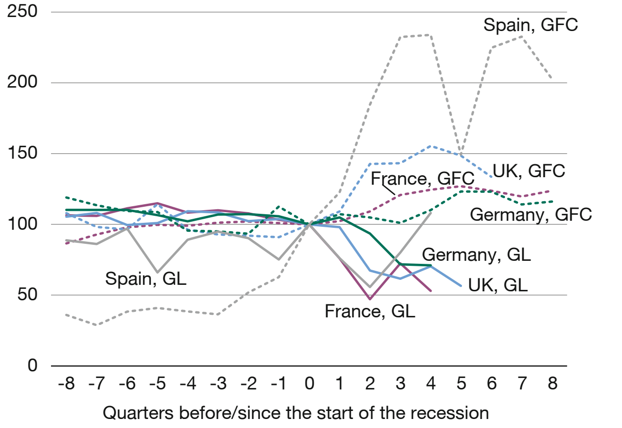

Note: GFC stands for global financial crisis; GL stands for great lockdown. French data are seasonally adjusted. No data for Northern Ireland are available before 2009Q4.

Sources: Authors' own calculations based on Banque de France and Insee, UK Insolvency Service, Statistisches Bundesamt and Instituto Nacional de Estadística.

them either much less or not at all. The most important exceptions are arts and entertainment, trade, travel and accommodation-related businesses. Travel has been particularly hard hit and its future prospects remain unclear. The number of EU flights remains 70% lower compared to 2019 (Eurostat, 2021).

However, strong effects in some sectors have not yet resulted in an increase in corporate insolvencies. Unlike the global financial crisis, the current "great lockdown" has in fact been associated with falling numbers of insolvencies (Figure 4). The data suggests that extraordinary fiscal support measures, both in terms of liquidity and capital (Anderson et al., 2021), combined with decisions to suspend and relax some insolvency notification requirements, are the main reasons for the falling rates. The European Systemic Risk Board (ESRB, 2021) warns of the big threat of a wave of insolvencies when current measures and support are phased out.

Other significant intra-EU divergence can be seen in the labour market, with the young and the less educated particularly affected (Figures 5 and 6). Generally, highly educated people have done well while for the less educated, there have been substantial employment losses. Moreover, the young have been disproportionally affected compared to older workers. There have even been increases in employment for those aged 55-65 (Grzegorczyk and Wolff, 2021). Women with low levels of education and women aged 15-24 appear to have suffered more than men (probably reflecting that they work in high-contact services that were strongly affected by lockdowns), while women aged 25-65 have been doing better in the labour market than men.

Education and age correlate strongly with income and wealth, and hence the labour market effects we have described suggest a widening of income inequalities.[3] From a survey of about 90 papers published in 2020-2021 on various aspects of inequality, Stantcheva (2021) concluded that COVID-19 has exacerbated existing inequalities across income groups, sectors, regions, gender, and between children from different backgrounds. Almeida et al. (2020) showed that in the absence of a policy response, disposable income inequality would have increased more.

School and university closures affect the most vulnerable parts of society. A study from the Organisation for Economic Co-operation and Development (Hanushek and Woessmann, 2020) suggested that students affected by school closures during the pandemic may experience 3%

3 See numerical scenarios for income inequality increases in the EU and globally in Darvas (2021).

Figure 5

**EU27 employment by educational level and gender**

index, 2019Q1=100

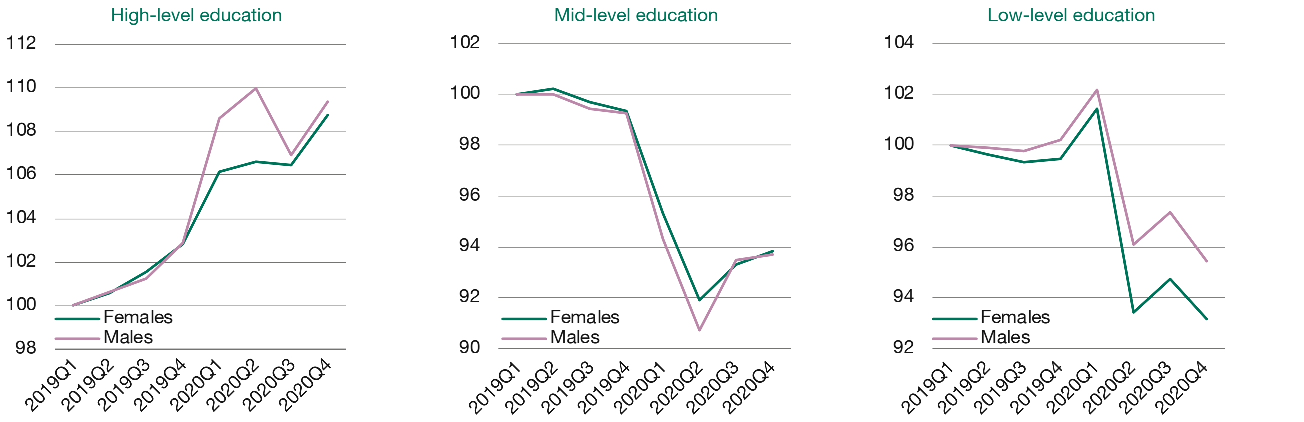

Note: Seasonally adjusted data.

Source: Authors' own calculations based on Eurostat's "Employment by sex, occupation and educational attainment level (1 000) [lfsq_egised]" dataset, 2021.

Figure 6

**EU27 employment by age and gender**

index, 2019Q1=100

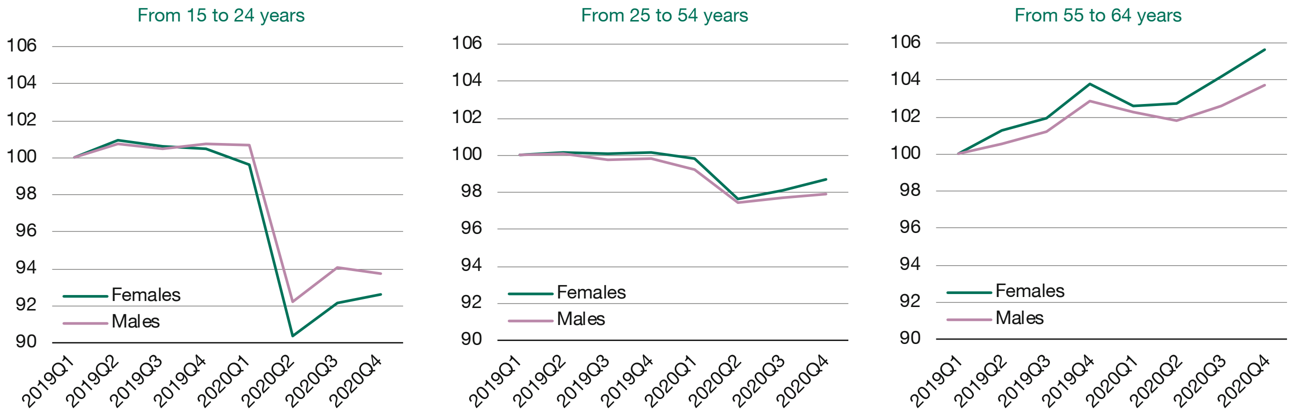

Note: Seasonally adjusted data.

Source: Authors' own calculations based on Eurostat's "Employment and activity by sex and age - quarterly data [lfsi_emp_q]" dataset, 2021.

lower lifetime incomes unless catch-up measures are put in place. This, they estimate, translates into a lower long-term level of output, because of the loss in productivity, in nations where education closures were most prominent. These numbers are worse for certain segments of society, particularly the less educated.

## Will there be structural shifts in our economies?

While the pandemic persists globally, some consumers may remain more cautious and adopt different behaviour to what was normal pre-pandemic. Given the still dramatic health crisis at the global level and the emergence of coronavirus variants, the situation is still very precarious. Globally, the pandemic is unlikely to be under control in 2022 and the virus may even become endemic (Phillips, 2021). This suggests that global travel patterns will not return to pre-pandemic levels soon and systematic screening for new variants will remain a vital measure to safeguard the local containment of the pandemic (Hellwig et al., 2021). Even within the EU, business travel is likely to remain at lower levels because of increased caution and because of the greater efficiency of online meetings.

However, there is also some evidence that consumers want to return to old patterns as soon as the health situation allows. Anecdotal evidence, which could indicate what a post-COVID-19 economy will look like, is emerging from countries that have almost completed their vaccine rollouts and have reopened earlier than the EU. One example is Israel, which has, at the time of writing, fully vaccinated around 60% of its total population. In Israel, credit-card spending has surged since the reopening in early March 2021 in particular of restaurants, hotels and clothing stores (spending was at first above pre-pandemic levels and then settled to about pre-pandemic levels). This suggests that consumer behaviour will tend to return to normal when permitted (Milhøj et al., 2021).

There are good reasons to believe that long-term productivity growth will increase. Based on a business survey in the US, the UK and five EU countries (France, Germany, Italy, Spain and Sweden), Mischke et al. (2021) estimated that there is potential for annual productivity growth to increase by about one percentage point up to 2024.

COVID-19 has forced firms to become more efficient. Firms that suddenly found themselves in prolonged shutdowns have had to optimise processes, cut down costs and become more efficient. They have had to become more innovative and to digitalise and automate as much as possible. Maqui and Morris (2020) showed that 75% of firms surveyed agreed that the pandemic helped make their business more efficient and resilient. Nine out of 10 firms had sped up the adoption of digital technology and automation.

The average level of productivity within sectors could mechanically increase as the least productive firms are forced to exit. This is known as the “cleansing effect”. Preliminary evidence provides some support, for example in France, where the average level of productivity has increased, albeit at a lower level of output (Hadjibeyli et al., 2021).

The prospect of teleworking will allow for greater flexibility, and arguably higher productivity. Maqui and Morris (2020) also found that 60% of surveyed respondents did not believe that teleworking reduces productivity. Many see advantages arising from greater overall flexibility, less commuting time and increased connectivity. Figure 7 shows the potential for increased teleworking by profession.

However, the overall net effect on productivity is uncertain. Bloom et al. (2020) showed that the efforts to deal with the pandemic have increased intermediate costs for UK firms. The authors estimated that productivity was reduced by up to 5% by the last quarter of 2020 and argued that the current management of the coronavirus pandemic may cause a reduction of 1% in the medium term in comparison to pre-pandemic levels due to less research and development expenditure as well as diverted time spent by managers to deal with pandemic-related issues.

Figure 7

**Teleworkability and actual teleworking in 2018 among employees by broad occupation group, EU27**

in %

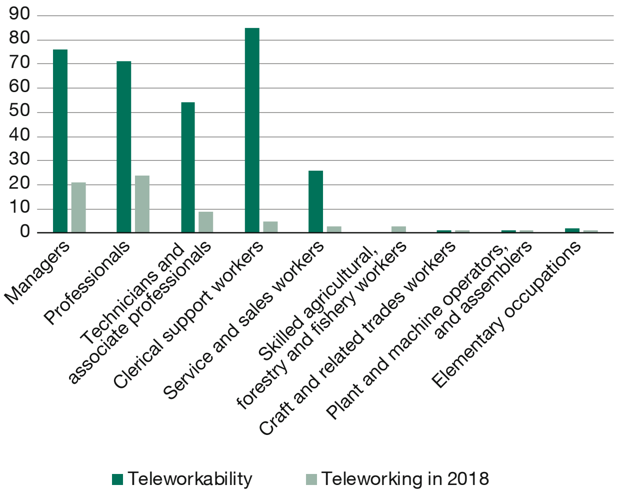

Note: Teleworkability refers to the proportion of employees who could telework.

Source: Figure 22 of Sostero et al. (2020).

A shortening of global value chains would increase costs and reduce efficiencies. Certain parts of production may be repatriated, reducing the length of global value chains, motivated by protectionism and the desire to increase resilience. Either way, this process will see an increase in overall costs. Arriola et al. (2020) estimated that a shortening of value chains will adversely affect competitiveness and temper productivity.[4] Whether and to what extent supply chains will be shortened remains, however, uncertain.

COVID-19 may have permanently affected market structures. Information and communication technology firms have seen increases in their market capitalisations. This has significantly increased their share in the Standard & Poor’s Index. To the extent that there is increased concentration, there could be negative productivity effects and negative effects on economic dynamism (Demertzis and Viegi, 2021).

4 OECD (2021) found the same based on model simulations.

Figure 8
**General government primary balance forecasts**

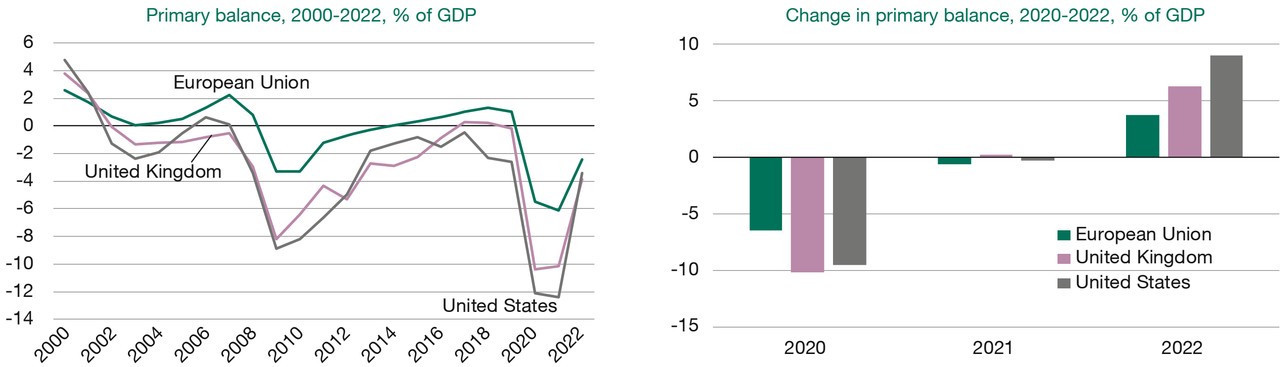

Note: The spending from Next Generation EU grants does not count as deficit of EU countries. The change in the primary balance results from both discretionary fiscal measures as well as automatic stabilisers.

Source: Authors' own calculations based on May 2021 European Commission forecast.

## Policy challenges ahead

Policymakers will face tremendous uncertainty in the next few years. The evolution of the pandemic remains the biggest risk to the global outlook, and policymakers need to prioritise the health emergency. But beyond the pandemic, behavioural change among individuals, and new work technologies and organisations may emerge. In addition, policymakers need to factor into their policy choices major political goals such as reducing greenhouse gas emissions.

### Fiscal policy orientation

The EU will reach pre-pandemic output levels later than the US, raising questions about the size and composition of fiscal support. The difference in growth performance can be explained by multiple factors, including differences in the management of the pandemic, different sectoral compositions, different market structures and levels of flexibility, and different fiscal policies. The difference in fiscal support during 2020 and 2021, however, is sizeable (Figure 8), with the scale of support in the US likely playing a role in its faster economic recovery.

We advise EU policymakers not to remove fiscal support too quickly. On the contrary, we see a justification for an additional short-term fiscal impulse in order to return to the late-2019 level of output earlier than currently forecast. If productivity growth is higher in the coming years thanks to the reorganisation of business processes, then more fiscal stimulus now should not create medium-term inflationary pressures (current IMF (2021) forecasts predict inflation in the euro area will be below 1.8% until 2026, which suggests there is some slack in the economy), nor should it raise debt sustainability concerns. To boost aggregate demand, and given the significant distributional consequences of the COVID-19 crisis and the loss of income in some categories of the population, fiscal support could in part take the form of targeted support to low-income households with low savings and a high propensity to consume.

Fiscal policymakers will need to move away gradually from supporting a broad set of companies towards broader demand support. Not only are corporate insolvencies at historically low levels, but the steady-state economy could look quite different from that of today. To allow for sectoral reallocation and reorganisation within sectors, not every firm can be kept alive forever by state support.[5] Fiscal policymakers should support the reallocation of productive factors by incentivising corporate investment and supporting the re-training and re-skilling of workers. Increasing the tax-deductibility of corporate investment, for example, would increase corporate investment and production. Fiscal incentives to upgrade digital infrastructures faster would also boost the recovery.

The fiscal policy framework needs to be reviewed if policymakers want to achieve a rapid green transition. Beyond necessary increases in the prices of greenhouse

5 Anderson et al. (2020) discussed the phases of the initial response and the phasing out of measures and their effects.

Figure 9

**Net fixed capital formation of the general government**

in % of GDP

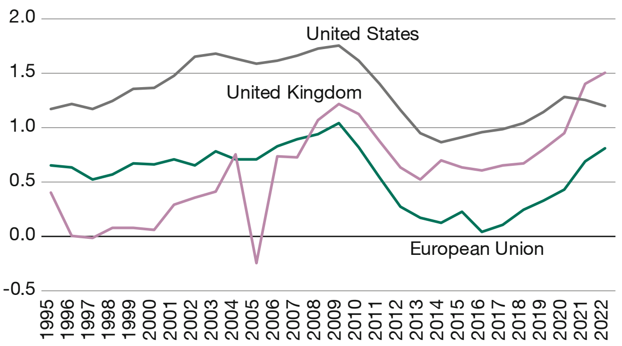

Note: European Union refers to the group of the current 27 member states. The estimated impact of Next Generation EU is incorporated in the forecast for the EU.

Source: Authors' own calculations based on May 2021 AMECO.

gas emissions, the EU will need to increase public investment in green infrastructure that the private sector cannot provide.[6] The EU has been falling behind other advanced economies in terms of public investment since around 2012 (Figure 9). While Next Generation EU will support public investment, EU net public investment as a share of GDP in 2022 is expected to remain well below the UK and US values, according to the May 2021 European Commission forecast. European fiscal rules tend to deter public investment, because investment is not privileged in the deficit rules.[7] While the costs of net public investment are incurred in a specific budget year and therefore need to be traded off against other spending or tax increases in order to meet fiscal targets, the benefits of such investments accrue over several years or even decades. It is likely, therefore, that the EU's fiscal rules have increased the short-term orientation in member state budgets and thereby reduced public investment. A review of fiscal rules is warranted with the aim of making them more encouraging to public green investment, for example, with some form of a green golden rule.

The EU's landmark recovery instrument, Next Generation EU (NGEU), and in particular the Recovery and Resilience Facility (RRF) regulation, supports a more medium-term orientation of fiscal policy as national recovery and resilience plans focus on green and digital transitions.[8] The RRF aims to address the various divergences between EU countries. The orientation towards green and digital spending, as well as the medium-term focus of the programme, while welcome, does not prevent short-term scarring risks in labour markets. National fiscal support programmes targeted at those most affected remain important to prevent scarring.

The reform components of the recovery plans are highly important. For example, Italy plans significant reforms to the judicial system and public administration. Structural weaknesses have been a major factor in divergent economic performances (Sapir, 2020), and such reforms have the potential to reinvigorate business activities.

NGEU can smooth the fiscal consolidation impacts once European fiscal rules are reactivated. If joint EU borrowing is not treated as national deficit and debt, then it will ease rules-based fiscal adjustment needs (Darvas and Wolff, 2021).[9]

Overall, fiscal policymakers need to focus increasingly on how resources are spent to improve economic performance and prevent scarring as well as on the progressivity of the taxation system. A short-term fiscal boost on its own is not enough to overcome the identified regional and structural divergences. Indiscriminate support for all companies may unnecessarily delay corporate restructuring. Good governance and administrative capacity are critical elements that determine the effectiveness of fiscal policies. Progressive tax systems are important in tackling income and wealth inequality and should be regularly reviewed.

## Insolvency law

Improving the efficiency of insolvency procedures will be crucial for speedy and effective recovery. Policymakers need to prepare for the wave of insolvencies that could quickly arrive once current safeguards are lifted.[10] The

6 For instance, this is the case for those enabling investments, such as smart grids and electric vehicles charging infrastructure, that are necessary to unleash further private investments in renewables and electric mobility.

7 The existing "investment clause" in the EU fiscal framework has a very limited scope, duration and is subject to strict conditions.

8 Our analysis shows that on average, the green transition accounts for about 43%, and the digital transition about 28% of the spending plans of those 23 countries that had submitted their plans by the time of writing this paper. Note that there is some overlap between green and digital projects (i.e. some projects are both green and digital).

9 The May 2021 European Commission forecast does not consider EU borrowing to finance NGEU grants as national debt and deficit, but NGEU loans to member states are considered as national debt (Box I.2.3 of European Commission (2021)). This suggests that the same approach might be adopted when EU fiscal rules are re-activated. However, it is unclear whether expenditures financed by NGEU loans will also be considered as national budget deficit. If spending financed by such loans does not benefit from special treatment in the EU fiscal framework, borrower countries will have to reduce their non-NGEU spending to make space for spending financed by NGEU loans, once the currently suspended fiscal rules are re-activated.

10 Claeys et al. (2021) provided more detail on insolvencies in the EU and the reform of insolvency regimes.

average recovery rate from insolvency procedures in the EU is 62 cents on the dollar, far below that of the UK (85 cents on the dollar) or the US (81 cents on the dollar) (World Bank, 2020). The European Banking Authority (2020) suggested that recovery rates in Europe might be even lower, with estimates ranging from 34 cents on the dollar for SMEs to 40 cents on the dollar for large companies. Insolvency procedures in the EU also take on average twice as long as in the UK and the US, and many frameworks in the EU favour liquidation over restructuring, thereby failing to protect remaining entrepreneurial value.

By reforming insolvency processes, policymakers can tackle critical impediments to economic growth in the post-COVID-19 recovery. In general, the focus should be on simplifying procedures, expanding court capacity and addressing the bureaucratic load. More specifically, ensuring that existing laws do not punish business failure excessively would strengthen market selection by facilitating firm exit and entry (Adalet McGowan et al., 2017; Peng et al., 2010). In addition, barriers to corporate restructuring should be reduced, for example by allowing early restructuring or creating cheaper procedures for smaller companies so they can avoid liquidation and the ensuing loss of business value.

At the EU level, policymakers should ensure the swift transposition into national legislation and effective implementation[11] of the Restructuring and Second Chance Directive (EU) 2019/1023, which aims to increase the coherence of insolvency procedures in EU countries and would introduce targeted measures to improve their efficiency. This would benefit the economy by promoting investment, innovation and economic growth, and would also represent an important step towards a capital markets union, notwithstanding that these structural changes are unlikely to have immediate effect. Obviously, the reform of the insolvency frameworks will take time but it is an important issue to tackle.

### Labour markets

COVID-19 has had unequal labour market effects, disadvantaging the young and less educated. The green transition is expected to have divergent labour market effects, calling for targeted policies. Empirical research also suggests that skill requirements and education levels are currently higher for green jobs than for non-green jobs (Griffin et al., 2019). Policymakers need to create specific programmes to support employment among the less qualified and the young, and to provide dedicated training opportunities.[12] Social policies, in particular a strong emphasis on education and life-long learning, will play a crucial role in the coming years to ensure that the benefits of the coming recovery, but also of the green and digital transitions (which can also have detrimental distributional effects), will be shared by all European citizens.

As teleworking becomes a more permanent feature of the EU's labour markets, it will be crucial to adapt social security and taxation systems in the context of the single market for labour. Teleworking could be a major driver of productivity in the coming years and could also be welfare enhancing and greener, for example, by allowing workers to commute less. An important question at the European level is how well social security systems are adapted to teleworking from other EU countries. Currently, significant obstacles exist, for example, when it comes to health insurance coverage. If the EU wants to reap the benefits of an integrated EU labour market, it will be necessary to review these approaches.

### Market structures

The EU should resist protectionist calls in the wake of the pandemic. While during the pandemic there have been instances of supply constraints, it would be a mistake to argue that reliance on purely EU supply chains would have resulted in better outcomes, even in narrow fields such as medical products. On the contrary, many of the EU's top companies rely on diversified global supply chains for cheap and high-quality production. While reviewing vulnerabilities and diversifying supplies may be advisable, a generalised protectionist stance will likely increase prices, reduce production capacity and slow down Europe's recovery, thereby contributing to divergence.

Rigorous competition policy enforcement and an integrated EU market have been beneficial for European convergence and growth. During the pandemic, extraordinary state subsidies were provided to companies across the EU. These subsidies were warranted given the mandatory sanitary measures. However, making state support permanent would undermine long-term growth performance. While targeted industrial policy measures can have positive growth effects in specific segments where market failures are particularly important, the EU will have to find the right balance between exiting the current support

11 At the time of writing, a number of countries have requested an extension until 2022 for the transposition of the Directive: Ireland, Italy, Latvia, the Netherlands, Portugal, Poland and Slovakia. Several more are expected to follow (Belgium, Sweden, Finland). See https://www.insol-europe.org/tracker-eu-directive-on-restructuring-and-insolvency.

12 Cameron et al. (2020) provided a detailed discussion in the context of the EU Just Transition Fund.

measures and ensuring market-driven growth. European industries became more concentrated already before the pandemic, a trend that could accelerate during the pandemic. Increased vigilance to identify market dominance in the digital and other sectors is warranted after COVID-19 to ensure more innovation and competition. As concerns the single market, restrictions on the free movement of people need to be removed as soon as there are no health-related justifications for maintaining them (rigorous testing may be necessary in light of the emergence of variants).

Finally, deep, liquid and integrated capital markets (and in particular a higher use of equity in corporate funding instead of debt) can help resolve debt overhangs after the pandemic and provide new impetus for growth. If insolvencies increase, it will be important to relieve stressed bank balance sheets rapidly. Capital markets can play a role in this. Re-energising the EU's capital markets union would also provide growth impetus by supporting risk capital. In the short to medium term, rigorous stress testing of bank balance sheets is advisable to detect and resolve obstacles to renewed lending activities.
